# Supplementary material for: Current Trends and Controversies in the Management of Warthin Tumor of the Parotid Gland
Source: Diagnostics (Basel). 2021 Aug 13;11(8):1467. doi: 10.3390/diagnostics11081467 (PMC8391156; doi:10.3390/diagnostics11081467)
Supplement: Supplementary file 1 [file diagnostics-11-01467-s001.zip › diagnostics-1323066-supplementary.pdf]

Supplementary material 1. References by alphabetic order.

1. Alkan U, Shkedy Y, Mizrachi A, Shpitzer T, Popovtzer A, Bachar G. Inflammation following invasive procedures for Warthin's tumour: A retrospective case series. Clin Otolaryngol. 2017 Dec;42(6):1241-1246. doi: 10.1111/coa.12857. Epub 2017 Mar 23. PMID: 28235157.
2. Allison DB, Smith AP, An D, Miller JA, Shafique K, Song S, Viswanathan K, Eykman E, Rao RA, Wiles A, Barkan GA, Nayar R, Fadda G, Powers CN, Rossi ED, Siddiqui MT, Ali SZ, Kholová I, Layfield LJ, Field A, Baloch Z, Maleki Z. Assessing the diagnostic accuracy for pleomorphic adenoma and Warthin tumor by employing the Milan System for Reporting Salivary Gland Cytopathology: An international, multi-institutional study. Cancer Cytopathol. 2021 Jan;129(1):43-52. doi: 10.1002/cncy.22339. Epub 2020 Aug 7.PMID: 32767837.
3. Alnoor FNU, Gandhi JS, Stein MK, Solares J, Gradowski JF. Prevalence of Lymphoid Neoplasia in a Retrospective Analysis of Warthin Tumor: A Single Institution Experience. Head Neck Pathol. 2020 Dec;14(4):944-950. doi: 10.1007/s12105-020-01161-z. Epub 2020 Apr 23. PMID: 32328910; PMCID: PMC7669968.
4. Aro K, Korpi J, Tarkkanen J, Mäkitie A, Atula T. Preoperative evaluation and treatment consideration of parotid gland tumors. Laryngoscope Investig Otolaryngol. 2020 Jul 20;5(4):694-702. doi: 10.1002/lio2.433. PMID: 32864441; PMCID: PMC7444776.
5. Auger SR, Kramer DE, Hardy B, Jandali D, Stenson K, Kocak M, Al-Khudari S. Functional outcomes after extracapsular dissection with partial facial nerve dissection for small and large parotid neoplasms. Am J Otolaryngol. 2021 Jan-Feb;42(1):102770. doi: 10.1016/j.amjoto.2020.102770. Epub 2020 Oct 22. PMID: 33188987.
6. Ballo MS, Shin HJ, Sneige N. Sources of diagnostic error in the fine-needle aspiration diagnosis of Warthin's tumor and clues to a correct diagnosis. *Diagn Cytopathol*. 1997;17(3):230-234. doi:10.1002/(sici)1097-0339(199709)17:3<230::aid-dc12>3.0.co;2-g
7. Barbara R-R, Pawaroo D, Beadsmoore C, Hujairi N, Newman D. Parotid incidentalomas on positron emission tomography: what is their clinical significance? Nucl Med Commun. 2019;40(3):264-269. doi:10.1097/MNM.0000000000000961
8. Ben-Dov T, Edelstein E, Nageris BI, Kassem F. Synchronous parotid and asopharyngeal Warthin's tumor: case report and literature review. Braz J Otorhinolaryngol. 2020 Dec;86 Suppl 1:44-47. doi: 10.1016/j.bjorl.2017.05.009. Epub 2017 Jun 27. PMID: 28711460.
9. Bieńkowski M, Kunc M, Iliszko M, Kuźniacka A, Studniarek M, Biernat W. MAML2 rearrangement as a useful diagnostic marker discriminating between Warthin tumour and Warthin-like mucoepidermoid carcinoma. Virchows Arch. 2020 Sep;477(3):393-400. doi: 10.1007/s00428-020-02798-5. Epub 2020 Mar 28. PMID: 32222825; PMCID: PMC7443186.
10. Borsetto D, Fussey JM, Cazzador D, Smith J, Ciorba A, Pelucchi S, Donà S, Boscolo-Rizzo P, Tomasoni M, Lombardi D, Nicolai P, Zanoletti E, Colangeli R, Emanuelli E, Osborne MS, Ahsan SF, Tofanelli M, Tirelli G, McNamara K, Liew L, Harrison K, Fassina A, Sarcognato S, Sharma N, Rao K, Pracy P, Nankivell P. The diagnostic value of cytology in parotid Warthin's tumors: international multicenter series. Head Neck. 2020 Mar;42(3):522-529. doi: 10.1002/hed.26032. Epub 2019 Nov 24.PMID: 31762130

11. Bothe C, Fernandez A, Quer M, Lop J. Parotid Incidentaloma Identified by Positron Emission / Computed Tomography : When to Consider Diagnoses Other than Warthin Tumor. Int arch otorhinolaryngol. 2015;19:112-115.
12. Bradley PJ, McGurk M. Incidence of salivary gland neoplasms in a defined UK population. Br J Oral Maxillofac Surg. 2013 Jul;51(5):399-403. doi: 10.1016/j.bjoms.2012.10.002. Epub 2012 Oct 24. PMID: 23103239.
13. Brennan PA, Ammar M, Matharu J. Contemporary management of benign parotid tumours - the increasing evidence for extracapsular dissection. Oral Dis. 2017 Jan;23(1):18-21. doi: 10.1111/odi.12518. Epub 2016 Jul 11. PMID: 27260128.
14. Chang YJ, Huang TY, Liu YJ, Chung HW, Juan CJ. Classification of parotid gland tumors by using multimodal MRI and deep learning. NMR Biomed. 2021 Jan;34(1):e4408. doi: 10.1002/nbm.4408. Epub 2020 Sep 4.PMID: 32886955.
15. Chen W, Zhu LN, Dai YM, Jiang JS, Bu SS, Xu XQ, Wu FY. Differentiation of salivary gland tumor using diffusion-weighted imaging with a fractional order calculus model. Br J Radiol. 2020 Sep 1;93(1113):20200052. doi:10.1259/bjr.20200052. Epub 2020 Jul 10. PMID: 32649236; PMCID: PMC7465841.
16. Chulam TC, Francisco ALN, Filho JG, Alves CAP, Kowalski LP. Warthin ' s tumour of the parotid gland : our experience. Acta Otorhinolaryngol Ital. 2013;33:393-397.
17. Coudert H, Mirafzal S, Dissard A, Boyer L, Montoriol PF. Multiparametric magnetic resonance imaging of parotid tumors: A systematic review. Diagn Interv Imaging. 2020 Sep 14:S2211-5684(20)30216-3. doi: 10.1016/j.diii.2020.08.002. Epub ahead of print. PMID: 32943368.
18. Dell'Aversana Orabona G, Bonavolontà P, Iaconetta G, Forte R, Califano L. Surgical management of benign tumors of the parotid gland: extracapsular dissection versus superficial parotidectomy--our experience in 232 cases. J Oral Maxillofac Surg. 2013 Feb;71(2):410-3. doi: 10.1016/j.joms.2012.05.003. Epub 2012 Aug 9. PMID: 22884114.Apr;49(3):237-8. doi: 10.1016/j.bjoms.2010.01.015.
19. Di Palma S, Simpson RH, Skálová A, Michal M. Metaplastic (infarcted) Warthin's tumour of the parotid gland: a possible consequence of fine needle aspiration biopsy. Histopathology. 1999;35(5):432-438. doi:10.1046/j.1365-2559.1999.035005432.x
20. Domenick NA, Johnson JT. Parotid tumor size predicts proximity to the facial nerve. Laryngoscope. 2011 Nov;121(11):2366-70. doi: 10.1002/lary.22335. PMID: 22020887.
21. El-Naggar AK, Takata JKCC, Grandis JR, Takata T, Slootweg PJ, On CIA for R. WHO Classification of Head and Neck Tumours, 9th Edition.; 2017.
22. Espinoza S, Felter A, Malinvaud D, Badoual C, Chatellier G, Siauve N, Halimi P. Warthin's tumor of parotid gland: Surgery or follow-up? Diagnostic value of a decisional algorithm with functional MRI. Diagn Interv Imaging. 2016 Jan;97(1):37-43. doi: 10.1016/j.diii.2014.11.024. Epub 2014 Dec 17. PMID: 25543869.
23. Ethunandan M, Pratt CA, Higgins B, Morrison A, Umar T, Macpherson DW. Factors influencing the occurrence of multicentric and ' recurrent ' Warthin ' s tumour : a cross sectional study. Int J Oral Maxillofac Surg. 2008;37:831-834. doi:10.1016/j.ijom.2008.05.001
24. Eveson JW, Cawson RA. Infarcted ('infected') adenolymphomas. A clinicopathological study of 20 cases. *Clin Otolaryngol Allied Sci*. 19989;14(3):205-210. doi:10.1111/j.1365-2273.1989.tb00362.x
25. Faquin WC, Rossi ED, Baloch Z, et al. *The Milan System for Reporting Salivary Gland Cytopathology*.; 2018.

26. Ferlito A, Bertino G, Rinaldo A, Mannarà GM, Devaney KO. A review of heterotopia and associated salivary gland neoplasms of the head and neck. J Laryngol Otol. 1999;113:299-303. doi:10.1017/s0022215100143841
27. Franzen AM, Coordes A, Franzen CK, Guenzel T. Are Multiple Tumors of the Parotid Gland Uncommon or Underestimated ? Anticancer Res. 2017;37:5263-5267. doi:10.21873/anticanres.11951
28. Franzen AM, Kaup C, Thomas F, Anja G. Increased incidence of Warthin tumours of the parotid gland : a 42- year evaluation. Eur Arch Oto-Rhino-Laryngology. 2018;275(10):2593-2598. doi:10.1007/s00405-018-5092-3
29. Gabelloni M, Faggioni L, Attanasio S, Vani V, Goddi A, Colantonio S, Germanese D, Caudai C, Bruschini L, Scarano M, Seccia V, Neri E. Can Magnetic Resonance Radiomics Analysis Discriminate Parotid Gland Tumors? A Pilot Study. Diagnostics (Basel). 2020 Nov 3;10(11):900. doi: 10.3390/diagnostics10110900.PMID: 33153140.
30. Gant TD, Hovey LM, Williams C. Surgical management of parotid gland tumors. Ann Plast Surg. 1981;6(5):389-392. doi:10.1097/00000637-198105000-00007
31. Gao M, Hao Y, Huang MX, Ma DQ, Chen Y, Luo HY, Gao Y, Cao ZQ, Peng X, Yu GY. Salivary gland tumours in a northern Chinese population: a 50-year retrospective study of 7190 cases. Int J Oral Maxillofac Surg. 2017 Mar;46(3):343-349. doi: 10.1016/j.ijom.2016.09.021. Epub 2016 Oct 19. PMID: 27769738.
32. García JJ, Hunt JL, Weinreb I, et al. Fluorescence in situ hybridization for detection of MAML2 rearrangements in oncocytic mucoepidermoid carcinomas: utility as a diagnostic test. *Hum Pathol*. 2011;42(12):2001-2009. doi:10.1016/j.humpath.2011.02.028
33. Goswami A, Zhang AJ, Vahidi S, Mettler T, Stewart J 3rd, Amin K. Oncocytic features in salivary duct carcinoma, a potential pitfall for misdiagnosis as Warthin tumor in fine needle aspiration specimens: A cytomorphologic analysis of 14 cases. Diagn Cytopathol. 2020 Jul;48(7):604-609. doi: 10.1002/dc.24426. Epub 2020 Apr 9. PMID: 32271502.
34. Hancox SH, Sinnott JD, Howlett DC. Re: Differentiation of pleomorphic adenoma and Warthin's tumour of the salivary gland: is long-to-short ratio a useful parameter? Clin Radiol. 2016 Apr;71(4):400-1. doi: 10.1016/j.crad.2015.11.023. Epub 2016 Feb 11. PMID: 26874659.
35. Hellquist H, Paiva-Correia A, Vander Poorten V, et al. Analysis of the Clinical Relevance of Histological Classification of Benign Epithelial Salivary Gland Tumours. Adv Ther. 2019;36(8):1950-1974. doi:10.1007/s12325-019-01007-3
36. Hung C, Kang B, Wang J-S, Lin Y-S. Ulcerative Warthin Tumor : A Case Report and Review of the Literature. Ear, Nose Throat J. 2020;386:1-4. doi:10.1177/0145561320957574
37. Iwai H, Yamashita T. Local excision procedure for Warthin's tumor of the parotid gland. Otolaryngol Head Neck Surg. 2005;132(4):577-580. doi:10.1016/j.otohns.2004.09.071.
38. Iro H, Zenk J. Role of extracapsular dissection in surgical management of benign parotid tumors. JAMA Otolaryngol Head Neck Surg. 2014 Aug;140(8):768-9. doi: 10.1001/jamaoto.2014.1218. PMID: 25033170
39. Ishibashi K, Ito Y, Masaki A, et al. Warthin-like Mucoepidermoid Carcinoma: A Combined Study of Fluorescence In Situ Hybridization and Whole-slide Imaging. *Am J Surg Pathol*. 2015;39(11):1479-1487. doi:10.1097/PAS.0000000000000507
40. Jechova A, Kuchar M, Novak S, Koucky V, Dostalova L, Zabrodsky M, Kalfert D, Plzak J. The role of fine-needle aspiration biopsy (FNAB) in Warthin tumour

diagnosis and management. Eur Arch Otorhinolaryngol. 2019 Oct;276(10):2941-2946. doi: 10.1007/s00405-019-05566-7. Epub 2019 Jul 18. PMID: 31321502.

41. Jin M, Fu J, Lu J, et al. Ultrasound-guided percutaneous microwave ablation of parotid gland adenolymphoma. Med. 2019;98(35):1-4.

42. Joo YH, Kim JP, Park JJ, Woo SH. Two-phase helical computed tomography study of salivary gland warthin tumors: a radiologic findings and surgical applications. Clin Exp Otorhinolaryngol. 2014 Sep;7(3):216-21. doi: 10.3342/ceo.2014.7.3.216. Epub 2014 Aug 1. PMID: 25177439; PMCID: PMC4135159.

43. Jung YJ, Han M, Ha EJ, Choi JW. Differentiation of salivary gland tumors through tumor heterogeneity: a comparison between pleomorphic adenoma and Warthin tumor using CT texture analysis. Neuroradiology. 2020 Nov;62(11):1451-1458. doi: 10.1007/s00234-020-02485-x. Epub 2020 Jul 3.PMID: 32621023

44. Kadletz L, Grasl S, Grasl MC, Perisanidis C, Erovic BM. Extracapsular dissection versus superficial parotidectomy in benign parotid gland tumors: The Vienna Medical School experience. Head Neck. 2017 Feb;39(2):356-360. doi: 10.1002/hed.24598. Epub 2016 Oct 5. PMID: 27704717.

45. Kadletz L, Grasl S, Perisanidis C, Grasl MC, Erovic BM. Rising incidences of Warthin's tumors may be linked to obesity : a single-institutional experience. Eur Arch Oto-Rhino-Laryngology. 2019;276(4):1191-1196. doi:10.1007/s00405-019-05319-6.

46. Kaleem A, Patel N, Alzahrani S, Hatoum H, Tursun R. Concurrent presence of secretory carcinoma and Warthin's tumor in ipsilateral parotid gland. Oral Oncol. 2020 Oct;109:104691. doi: 10.1016/j.oraloncology.2020.104691. Epub 2020 Apr 21. PMID: 32331963.

47. Kato H, Kanematsu M, Mizuta K, Aoki M, Hirose Y. Spontaneous infarction of Warthin's tumor: imaging findings simulating malignancy. Jpn J Radiol. 2012 May;30(4):354-7. doi: 10.1007/s11604-012-0056-9. Epub 2012 Feb 17.PMID: 22350592

48. Kato H, Fujimoto K, Matsuo M, Mizuta K, Aoki M. Usefulness of diffusion-weighted MR imaging for differentiating between Warthin's tumor and oncocytoma of the parotid gland. Jpn J Radiol. 2017 Feb;35(2):78-85. doi: 10.1007/s11604-016-0608-5. Epub 2017 Jan 10. PMID: 28074380.

49. Kim TY, Lee Y. Contrast-enhanced Multi-detector CT Examination of Parotid Gland Tumors: Determination of the Most Helpful Scanning Delay for Predicting Histologic Subtypes. J Belg Soc Radiol. 2019 Jan 3;103(1):2. doi: 10.5334/jbsr.1596. PMID: 30623171; PMCID: PMC6319312.

50. Kim JE, Kim TG. Squamous cell carcinoma arising from Warthin's tumor in the parotid gland. BJR Case Rep. 2019 Nov 15;5(4):20190032. doi: 10.1259/bjrcr.20190032. PMID: 31938564; PMCID: PMC6945251.

51. Klijanienko J, Vielh P. Fine-needle sampling of salivary gland lesions. II. Cytology and histology correlation of 71 cases of Warthin's tumor (adenolymphoma). *Diagn Cytopathol.* 1997;16(3):221-225. doi:10.1002/(sici)1097-0339(199703)16:3<221::aid-dc5>3.0.co;2-i

52. Klussmann JP, Wittekindt C, Preuss SF, Attab A Al, Schroeder U, Guntinas-Lichius O. High risk for bilateral Warthin tumor in heavy smokers – review of 185 cases. Acta Otolaryngol. 2006;126(11):1213-1217. doi:10.1080/00016480600740605

53. Laccourreye O LC. Warthin's tumour: Not so benign! Eur Ann Otorhinolaryngol Head Neck Dis. 2020;137:449. doi:10.1016/j.anorl.2020.05.003.

54. Lacko M, Voogd AC, van de Goor RC, Roelofs HM, Te Morsche RH, Bouvy ND, Peters WH, Manni JJ. Genetic polymorphisms in UDP-glucuronosyltransferase 1A6 and 1A7 and the risk for benign Warthin's tumors of the parotid gland. Head Neck.

2016 Apr;38 Suppl 1:E717-23. doi: 10.1002/hed.24077. Epub 2015 Jul 29. PMID: 25899702.

55. Lee DH, Yoon TM, Lee JK, Lim SC. Surgical treatment outcomes of patients with bilateral warthin tumors in the parotid gland. Indian J Otolaryngol Head Neck Surg. 2014 Jan;66(Suppl 1):303-6. doi: 10.1007/s12070-012-0499-4. Epub 2012 Feb 1. PMID: 24533403; PMCID: PMC3918311.

56. Lee DH, Yoon TM, Lee JK, Lim SC. Extracapsular dissection for Warthin tumor in the tail of parotid gland. Acta Otolaryngol. 2017;137(9):1007-1009. doi:10.1080/00016489.2017.1318221

57. Lee DH, Yoon MT, Lee JK, Lim SC. Surgical treatment strategy in Warthin tumor of the parotid gland. Braz J Otorhinolaryngol. 2020;85(5):546-550. doi:10.1016/j.bjorl.2018.04.004

58. Leverstein H, Van der Wal JE, Tiwari RM, Van der Waal I, Snow GB. Results of the surgical management and histopathological evaluation of 88 parotid gland Warthin's tumours. Clin Otolaryngol Allied Sci. 1997;22(6):500-503. doi:10.1046/j.1365-2273.1997.00054.x

59. Liu Y, Zheng J, Lu X, Wang Y, Meng F, Zhao J, Guo C, Yu L, Zhu Z, Zhang T. Radiomics-based comparison of MRI and CT for differentiating pleomorphic adenomas and Warthin tumors of the parotid gland: a retrospective study. Oral Surg Oral Med Oral Pathol Oral Radiol. 2021 Jan 15:4555. doi: 10.1016/j.oooo.2021.01.014. Online ahead of print.PMID: 33602604

60. Liu YJ, Lee YH, Chang HC, Chung HW, Wang CW, Juan CH, Chu YH, Lee JC, Juan
a. CJ. Imaging quality of PROPELLER diffusion-weighted MR imaging and its diagnostic performance in distinguishing pleomorphic adenomas from Warthin tumors of the parotid gland. NMR Biomed. 2020 May;33(5):e4282. doi: 10.1002/nbm.4282. Epub 2020 Mar 2. PMID: 32124504.

61. Luers JC, Guntinas-Lichius O, Klussmann JP, Küsgen C, Beutner D, Grosheva M. The incidence of Warthin tumours and pleomorphic adenomas in the parotid gland over a 25-year period. Clin Otolaryngol. 2016;41:793-798.

62. Maiorano E, Muzio L Lo, Favia G, Piattelli A. Warthin ' s tumour : a study of 78 cases with emphasis on bilaterality , multifocality and association with other malignancies. Oral Oncol. 2002;38:35-40.

63. Mamidi IS, Lee E, Benito DA, et al. Ultrasound-guided ethanol sclerotherapy for non-surgical treatment of Warthin' s tumor. Am J Otolaryngol Neck Med Surg. 2021;42(1):102813. doi:10.1016/j.amjoto.2020.102813

64. Mann L, Crosher R, Steel C. Warthin ' s tumour – Resolution following FNA. Int J Surg Case Rep. 2014;5(8):471-472. doi:10.1016/j.ijscr.2014.04.028

65. Mantsopoulos K, Goncalves M, Koch M. Extracapsular Dissection for Warthin Tumors Despite the Risk of Ipsilateral Metachronous Occurrence. Laryngoscope. 2018;128:2521-2524. doi:10.1002/lary.27166

66. Mantsopoulos K, Goncalves M, Koch M, Traxdorf M, Schapher M, Iro H. Going beyond extracapsular dissection in cystadenolymphomas of the parotid gland. Oral Oncol. 2020;88(November 2018):168-171. doi:10.1016/j.oraloncology.2018.12.001

67. Mantsopoulos K, Koch M, Goncalves M, Iro H. Investigation of the surgical strategies for unilateral multifocal cystadenolymphomas of the parotid gland. Oral Oncol. 2020;82(February 2018):176-180. doi:10.1016/j.oraloncology.2018.05.022

68. Mantsopoulos K, Goncalves M, Koch M, Iro H. Watchful waiting in carefully selected metachronous cystadenolymphomas of the parotid gland: a reliable option? Brazilian J Oral Maxillofac Surg. 2019;57(5):425-429. doi:10.1016/j.bjoms.2018.12.018.

69. Mantsopoulos K, Psychogios G, Agaimy A, Künzel J, Zenk J, Iro H, Bohr C. Inflamed benign tumors of the parotid gland: diagnostic pitfalls from a potentially misleading entity. Head Neck. 2015 Jan;37(1):23-9. doi: 10.1002/hed.23541. Epub 2014 Feb 1. PMID: 24488708.
70. Matsuda E, Fukuhara T, Donishi R, Kawamoto K, Hirooka Y, Takeuchi H. Usefulness of a Novel Ultrasonographic Classification Based on Anechoic Area Patterns for Differentiating Warthin Tumors from Pleomorphic Adenomas of the Parotid Gland. Yonago Acta Medica 2017;60:220–226 doi: 10.24563/yam.2017.12.002
71. McMullen CP, Smith R V, Ow TJ, Tassler A, Schiff BA. Minimal Margin Extracapsular Dissection: A Viable Alternative Technique for Benign Parotid Lesions? Ann Otol Rhinol Laryngol. 2016;125(11):912-917. doi:10.1177/0003489416661344
72. Miao LY, Xue H, Ge HY, Wang JR, Jia JW, Cui LG. Differentiation of pleomorphic adenoma and Warthin's tumour of the salivary gland: is long-to-short diameter ratio a useful parameter? Clin Radiol. 2015 Nov;70(11):1212-9. doi: 10.1016/j.crad.2015.06.085. Epub 2015 Jul 26. PMID: 26216455.
73. Mikaszewski B, Markiet K, Smugała A, Stodulski D, Szurowska E, Stankiewicz C. Clinical and demographic data improve diagnostic accuracy of dynamic contrast-enhanced and diffusion-weighted MRI in differential diagnostics of parotid gland tumors. Oral Oncol. 2020 Dec;111:104932. doi:10.1016/j.oraloncology.2020.104932. Epub 2020 Jul 30. PMID: 32739792.
74. Moore FO, Abdel-Misih RZ, Berne JD, Zieske AW, Rana NR, Ryckman JG. Poorly differentiated carcinoma arising in a Warthin's tumor of the parotid gland: pathogenesis, histopathology, and surgical management of malignant Warthin's tumors. Am Surg. 2007 Apr;73(4):397-9. PMID: 17439037.
75. McLean-Holden AC, Bishop JA. Low Molecular Weight Cytokeratin Immunohistochemistry Reveals That Most Salivary Gland Warthin Tumors and Lymphadenomas Arise in Intraparotid Lymph Nodes. Head Neck Pathol. 2020 Aug 31. doi: 10.1007/s12105-020-01215-2. Epub ahead of print. PMID: 32865726.
76. Moeller K, Esser D, Boeger D, Buentzel J, Hoffmann K, Jecker P, Mueller A, Radtke G, Piesold JU, Schultze-Mosgau S, Finkensieper M, Bitter T, Guntinas-Lichius O. Parotidectomy and submandibulectomy for benign diseases in Thuringia, Germany: a population-based study on epidemiology and outcome. Eur Arch Otorhinolaryngol. 2013 Mar;270(3):1149-55. doi: 10.1007/s00405-012-2225-y. Epub 2012 Oct 17. PMID: 23073736.
77. Nardi C, Tomei M, Pietragalla M, Calistri L, Landini N, Bonomo P, Mannelli G, Mungai F, Bonasera L, Colagrande S. Texture analysis in the characterization of parotid salivary gland lesions: A study on MR diffusion weighted imaging. Eur J Radiol. 2021 Mar;136:109529. doi: 10.1016/j.ejrad.2021.109529. Epub 2021 Jan 7.PMID: 33453571
78. Nguyen VX, Nguyen BD, Ram PC. Bilateral and multifocal Warthin's tumors of parotid glands: PET/CT imaging. Clin Nucl Med. 2012 Feb;37(2):175-7. doi: 10.1097/RLU.0b013e318238f244. PMID: 22228345.
79. Onken AM, Nishino M. Assessing the diagnostic accuracy for pleomorphic adenoma and Warthin tumor by employing the Milan System for Reporting Salivary Gland Cytopathology: An international, multi-institutional study. Cancer Cytopathol. 2020 Dec 1. doi: 10.1002/cncy.22392. Online ahead of print.PMID: 33259150
80. Orabona GD, Abbate V, Piombino P, Romano A, Schonauer F, Iaconetta G, Salzano G, Farina F, Califano L. Warthin's tumour: Aetiopathogenesis dilemma, ten years of

our experience. J Craniomaxillofac Surg. 2015 May;43(4):427-31. doi: 10.1016/j.jcms.2014.11.019. Epub 2014 Nov 28. PMID: 25814196.

81. Ozturk K, Ozturk A, Turhal G, Kaya I, Akyildiz S, Uluoz U. Comparative outcomes of extracapsular dissection and superficial parotidectomy. Acta Otolaryngol. 2019 Dec;139(12):1128-1132. doi: 10.1080/00016489.2019.1669821. Epub 2019 Sep 27. PMID: 31560243.

82. Palmer TJ, Gleeson MJ, Eveson JW, Cawson RA. Oncocytic adenomas and oncocytic hyperplasia of salivary glands: a clinicopathological study of 26 cases. *Histopathology*. 1990;16(5):487-493. doi:10.1111/j.1365-2559.1990.tb01549.x

83. Pantanowitz L, Thompson LDR, Rossi ED. Diagnostic Approach to Fine Needle Aspirations of Cystic Lesions of the Salivary Gland. Head Neck Pathol. 2018 Dec;12(4):548-561. doi: 10.1007/s12105-018-0904-8. Epub 2018 Mar 9. PMID: 29524082; PMCID: PMC6232202.

84. Patel DK, Ahmad Z, Morton RP. Partial Superficial Parotidectomy With Retrograde Dissection of the Facial Nerve for Clinically "Benign" Parotid Tumors. Ann Otol Rhinol Laryngol. 2016 Oct;125(10):808-14. doi: 10.1177/0003489416655352. Epub 2016 Jun 28. PMID: 27354214.

85. Patel DK, Morton RP. Demographics of benign parotid tumours : Warthin's tumour versus other benign salivary tumours. Acta Otolaryngol. 2016;136(1):83-86.

86. Psychogios G, Bohr C, Constantinidis J, et al. Review of surgical techniques and guide for decision making in the treatment of benign parotid tumors. Eur Arch Otorhinolaryngol. 2021;278(1):15-29. doi:10.1007/s00405-020-06250-x

87. Psychogios G, Vlastos I, Thölken R, Zenk J. Warthin ' s tumour seems to be the most common benign neoplasm of the parotid gland in Germany. Eur Arch Oto-Rhino-Laryngology. 2020;277(7):2081-2084. doi:10.1007/s00405-020-05894-z

88. Pujol-Olmo A, Mirapeix RM, Sañudo-Tejero JR, Quer-Agustí M. Description and relationships of the parotid gland levels proposed by the European Salivary Gland Society staging system: an anatomical study. Surg Radiol Anat. 2020; 42(9):1101-1107. doi: 10.1007/s00276-020-02483-x. Epub 2020 May 5.PMID: 32372113

89. Quer M, Marchal F, Vander Poorten V, et al. Classification of parotidectomies: a proposal of the European Salivary Gland Society. Eur Arch Oto-Rhino-Laryngology. 2016;273(10):3307-3312. doi:10.1007/s00405-016-3916-6

90. Quer M, Vander Poorten V, Takes RP, Silver CE, Boedeker C, Bree R De. Surgical options in benign parotid tumors: a proposal for classification. Eur Arch Otorhinolaryngol. 2017;274(11):3825-3836. doi:10.1007/s00405-017-4650-4

91. Rassekh CH, Cost JL, Hogg JP, Hurst MK, Marano GD, Ducatman BS. Positron emission tomography in Warthin's tumor mimicking malignancy impacts the evaluation of head and neck patients. Am J Otolaryngol. 2015 Mar-Apr;36(2):259-63. doi: 10.1016/j.amjoto.2014.11.008. Epub 2014 Nov 27.PMID: 25523505

92. Reddy V, Thangarajah T, Castellanos-Arango F, Panarese A. Conservative management of Warthin tumour. J Otolaryngol Head Neck Surg. 2008;37(5):744-749.

93. Ruohoalho J, Mäkitie AA, Aro K, et al. Complications after surgery for benign parotid gland neoplasms: A prospective cohort study. Head Neck. 2017;39(1):170-176. doi:10.1002/hed.24496.

94. Ryoo I, Suh S, Lee YH, Seo HS, Seol HY, Woo JS, Kim SC. Vascular Pattern Analysis on Microvascular Sonography for Differentiation of Pleomorphic Adenomasand Warthin Tumors of Salivary Glands. J Ultrasound Med. 2018 Mar;37(3):613-620. doi: 10.1002/jum.14368. Epub 2017 Sep 8. PMID: 28885737.

95. Sabour S. Cytology in parotid Warthin's tumors: Methodological issues on diagnostic value to avoid misinterpretation. Head Neck. 2020 Aug;42(8):2213-2214. doi: 10.1002/hed.26112. Epub 2020 Feb 28. PMID: 32109334.
96. Sagiv D, Witt RL, Glikson E, Mansour J, Slonimsky G, Talmi YP. Warthin tumor within the superficial lobe of the parotid gland : a suggested criterion for diagnosis. Eur Arch Oto-Rhino-Laryngology. 2017;274(4):1993-1996. doi:10.1007/s00405-016-4436-0
97. Sarioglu O, Sarioglu FC, Akdogan AI, Kucuk U, Arslan IB, Cukurova I, Pekcevik Y. MRI-based texture analysis to differentiate the most common parotid tumours. Clin Radiol. 2020 Nov;75(11):877.e15-877.e23. doi: 10.1016/j.crad.2020.06.018. Epub 2020 Jul 20. PMID: 32703544.
98. Schwalje AT, Uzelac A, Ryan WR. Growth rate characteristics of Warthin’s tumours of the parotid gland. Int J Oral Maxillofac Surg. 2015;44(12):1474-1479. doi:10.1016/j.ijom.2015.07.019
99. Seok J, Jeong WJ, Ahn SH, Jung YH. The growth rate and the positive prediction of needle biopsy of clinically diagnosed Warthin's tumor. Eur Arch Otorhinolaryngol. 2019 Jul;276(7):2091-2096. doi: 10.1007/s00405-019-05493-7. Epub 2019 Jun 5.PMID: 31165929
100. Shkedy Y, Alkan U, Mizrachi A, Shochat T, Dimitstein O, Morgenstern S, Shpitzer T, Bachar G. Fine-needle aspiration cytology for parotid lesions, can we avoid surgery?. Clin Otolaryngol. 2018 Apr;43(2):632-637. doi: 10.1111/coa.13038. Epub 2017 Dec 21.PMID: 29178364
101. Skálová A, Vanecek T, Simpson RHW, et al. CRTC1-MAML2 and CRTC3-MAML2 fusions were not detected in metaplastic Warthin tumor and metaplastic pleomorphic adenoma of salivary glands. *2013*. 37AD;11(1743-50). doi:10.1097/PAS.0000000000000065
102. Slater LJ. Parotid necrotizing sialometaplasia vs infarcted Warthin tumour. Dentomaxillofac Radiol. 2015;44(4):20140392. doi: 10.1259/dmfr.20140392. Epub 2015 Jan 7. PMID: 25564889; PMCID: PMC4628433.
103. Ślęzak A, Kaźmierczak W, Kaźmierczak H. Results of cystadenolymphoma treatment of superficial parotid gland. Otolaryngol Pol. 2018 Sep 10;72(6):23-30. doi: 10.5604/01.3001.0012.4667. PMID: 30647197.
104. So T, Sahovaler A, Nichols A, Fung K, Yoo J, Weir MM, MacNeil SD. Utility of clinical features with fine needle aspiration biopsy for diagnosis of Warthin tumor. J Otolaryngol Head Neck Surg. 2019 Aug 29;48(1):41. doi: 10.1186/s40463-019-0366-3.PMID: 31464652
105. Sood N, Borah P. Warthins tumor: Cyto histological spectrum with emphasis on diagnostic difficulties. Diagn Cytopathol. 2018 Jul;46(7):613-619. doi: 10.1002/dc.23896. Epub 2018 Feb 5. PMID: 29399982.
106. Stryjewska-Makuch G, Kolebacz B, A Janik M, Wolnik A. Increase in the incidence of parotid gland tumors in the years 2005-2014. Otolaryngol Pol. 2017;71(2):29-34. doi:10.5604/01.3001.0009.8412.
107. Sučić M, Ljubić N, Perković L, Ivanović D, Pažanin L, Sučić Radovanović T, Župnić-Krmek D, Knežević F. Cytopathology and diagnostics of Warthin's tumour. Cytopathology. 2020 May;31(3):193-207. doi: 10.1111/cyt.12830. PMID: 32259367.
108. Sun Q, Ma C, Dong M, Jiang M, Tao X. Effects of region of interest sizes on apparent diffusion coefficient measurements of pleomorphic adenoma, Warthin tumor, and normal parotid parenchyma. Quant Imaging Med Surg. 2019 Apr;9(4):681-690. doi: 10.21037/qims.2019.04.11. PMID: 31143659; PMCID: PMC6511721.
109. Taylor TR, Cozens NJA, Robinson I. Warthin’s tumour: a retrospective case series. Brazilian J Radiol. 2009;82(983):916-919. doi:10.1259/bjr/30175196

110. Teymoortash A. Back to the roots of Warthin's tumor of the parotid gland. Eur Arch Otorhinolaryngol. 2013 Sep;270(9):2397-402. doi: 10.1007/s00405-012-2309-8. Epub 2012 Dec 11. PMID: 23229644.
111. Teymoortash A, Bohne F, Jonsdottir T, Hoch S, Eivazi B, Roessler M, Werner JA, Mandic R. Human papilloma virus (HPV) is not implicated in the etiology of Warthin's tumor of the parotid gland. Acta Otolaryngol. 2013 Sep;133(9):972-6. doi: 10.3109/00016489.2013.797603. PMID: 23944949.
112. Teymoortash A, Krasnewicz Y, Werner JA. Clinical features of cystadenolymphoma (Warthin’s tumour) of the parotid gland: a retrospective comparative study of 96 cases. Oral Oncol. 2006;42(6):569-573. doi:10.1016/j.oraloncology.2005.10.017.
113. Thangarajah T, Reddy VM, Panarese A. Current controversies in the management of Warthin tumour. Postgrad Med J. 2009;85:3-8. doi:10.1136/pgmj.2008.071282
114. Thompson LD, Wenig BM, Ellis GL. Oncocytomas of the submandibular gland. A series of 22 cases and a review of the literature. *Cancer*. 1996;78(11):2281-2287. doi:10.1002/(sici)1097-0142(19961201)78:11<2281::aid-cncr3>3.0.co;2-q
115. Tretiakow D, Stodulski D, Skorek A. Regarding the concurrent presence of secretory carcinoma and Warthin's tumor in the ipsilateral parotid gland. Oral Oncol. 2020 Oct;109:104763. doi: 10.1016/j.oraloncology.2020.104763. Epub 2020 May 7. PMID: 32389539.
116. Tunç O, Gönüldaş B, Arslanhan Y, Kanlıkama M. Change in Warthin ’ s tumor incidence : a 20 - year joinpoint trend analysis. Eur Arch Oto-Rhino-Laryngology. 2020;277(12):3431-3434. doi:10.1007/s00405-020-06081-w.
117. Tung YC, Luo SD, Su YY, Chen WC, Chen HL, Cheng KL, Lin WC. Evaluation of Outcomes following Radiofrequency Ablation for Treatment of Parotid Tail Warthin Tumors. J Vasc Interv Radiol. 2019 Oct;30(10):1574-1580. doi: 10.1016/j.jvir.2019.04.031. Epub 2019 Aug 27. PMID: 31471194.
118. Üstün F, Taştekin E, Taş A, Altun GD. The Clinical Significance of Incidental Parotid Uptake in a PET/CT Study: A Diagnostic Algorithm. Curr Med Imaging Rev. 2019;15(3):326-333. doi:10.2174/1573405614666171213160244
119. Vaz SC, Marques A, Lourenço J, Galzerano A. Warthin Tumor Incidentally Detected on PET/CT Showing Both 68Ga-DOTANOC and 18F-FDG Uptake. Clin Nucl Med. 2021;46(2):118-120. doi:10.1097/RLU.0000000000003291.
120. Veder LL, Kerrebijn JD, Smedts FM, den Bakker MA. Diagnostic accuracy of fine-needle aspiration cytology in Warthin tumors. Head Neck. 2010 Dec;32(12):1635-40. doi: 10.1002/hed.21382. PMID: 20848407.
121. Vlantis AC, Ng SK, Mak CK, Cheung JM, Chan AB, van Hasselt CA. If cytology of Warthin tumor is accurate, can management be conservative? Ear, Nose Throat J. 2016;95(4-5):185-188.
122. Wang CW, Chu YH, Chiu DY, Shin N, Hsu HH, Lee JC, Juan CJ. JOURNAL CLUB: The Warthin Tumor Score: A Simple and Reliable Method to Distinguish Warthin Tumors From Pleomorphic Adenomas and Carcinomas. AJR Am J Roentgenol. 2018 Jun;210(6):1330-1337. doi: 10.2214/AJR.17.18492. Epub 2018 Apr 18.PMID: 29667889.
123. Warthin AS. Papillary Cystadenoma Lymphomatosum. A Rare Teratoid of the Parotid Region. J Cancer Res. 1929;13(2):116-125.
124. Williamson JD, Simmons BH, el-Naggar A, Medeiros LJ. Mucoepidermoid carcinoma involving Warthin tumor. A report of five cases and review of the literature. Am J Clin Pathol. 2000 Oct;114(4):564-70. doi: 10.1309/GUT1-F58A-V4WV-0D8P. PMID: 11026102.

125. White CK, Williams KA, Rodriguez-Figueroa J, Langer CJ. Warthin's tumors and their relationship to lung cancer. Cancer Invest. 2015 Jan;33(1):1-5. doi: 10.3109/07357907.2014.979365. Epub 2014 Dec 4.PMID: 25472027
126. Witt RL, Iacocca M, Gerges F. Contemporary diagnosis and management of Warthin's tumor. Del Med J. 2015;87(1):13-16.
127. Witt RL. Extracapsular Dissection with Facial Nerve Dissection for Benign Parotid Tumors. Otolaryngol Head Neck Surg. 2016 Mar;154(3):572-4. doi: 10.1177/0194599815627818. Epub 2016 Feb 16. PMID: 26884366.
128. Wong WK, Shetty S. The extent of surgery for benign parotid pathology and its influence on complications: A prospective cohort analysis. Am J Otolaryngol. 2018 Mar-Apr;39(2):162-166. doi: 10.1016/j.amjoto.2017.11.015. Epub 2017 Nov 29. PMID: 29246390.
129. Woodhouse NR, Gok G, Howlett DC, Ramesar K. Warthin's tumour and facial nerve palsy: an unusual association. Br J Oral Maxillofac Surg. 2011
130. Xie S, Wang K, Xu H, Hua RX, Li TZ, Shan XF, Cai ZG. PRISMA-Extracapsular Dissection Versus Superficial Parotidectomy in Treatment of Benign Parotid Tumors: Evidence From 3194 Patients. Medicine (Baltimore). 2015 Aug;94(34):e1237. doi: 10.1097/MD.0000000000001237. PMID: 26313768; PMCID: PMC4602923.
131. Xu W, Lu H, Zhu Y, Ruan M, Zhang C, Yang W, Liu S. Warthin's tumour in oral and maxillofacial regions: an 18-year retrospective study of 1084 cases in an eastern-Chinese population. Int J Oral Maxillofac Surg. 2018 Jul;47(7):913-917. doi: 10.1016/j.ijom.2018.03.013. Epub 2018 Apr 5. PMID: 29627151
132. Yabuuchi H, Kamitani T, Sagiyama K, Yamasaki Y, Hida T, Matsuura Y, Hino T, Murayama Y, Yasumatsu R, Yamamoto H. Characterization of parotid gland tumors: added value of permeability MR imaging to DWI and DCE-MRI. Eur Radiol. 2020 Dec;30(12):6402-6412. doi: 10.1007/s00330-020-07004-3. Epub 2020 Jul 1. PMID:32613285.
133. Yamada S, Matsuo T, Fujita S, Suyama K, Yamaguchi A, Mizuno A. Mucoepidermoid carcinoma arising in Warthin's tumor of the parotid gland. Pathol Int. 2002 Oct;52(10):653-6. doi: 10.1046/j.1440-1827.2002.01408.x. PMID: 12445138.
134. Yamamoto T, Kimura H, Hayashi K, Imamura Y, Mori M. Pseudo-continuous arterial spin labeling MR images in Warthin tumors and pleomorphic adenomas of the parotid gland: qualitative and quantitative analyses and their correlation with histopathologic and DWI and dynamic contrast enhanced MRI findings. Neuroradiology. 2018 Aug;60(8):803-812. doi: 10.1007/s00234-018-2046-9. Epub 2018 Jul 2. PMID: 29968073.
135. Yariv O, Popovtzer A, Wasserzug O, Neiderman NC, Halperin D, Lahav Y, Lahav G, Yehuda M. Usefulness of ultrasound and fine needle aspiration cytology of major salivary gland lesions. Am J Otolaryngol. 2020 Jan-Feb;41(1):102293. doi: 10.1016/j.amjoto.2019.102293. Epub 2019 Sep 10. PMID: 31732301.
136. Yoo GH, Eisele DW, Askin FB, Driben JS, Johns ME. Warthin's tumor: a 40-year experience at The Johns Hopkins Hospital. Laryngoscope. 1994 Jul;104(7):799-803. doi: 10.1288/00005537-199407000-00004. PMID: 8022240.
137. Yu C, Song Z, Xiao Z, Lin Q, Dong X. Mucoepidermoid carcinoma arising in Warthin's tumor of the parotid gland: Clinicopathological characteristics and immunophenotypes. Sci Rep. 2016 Jul 15;6:30149. doi: 10.1038/srep30149. PMID: 27417276; PMCID: PMC4945913.
138. Yu Y, Zhang WB, Soh HY, Sun ZP, Yu GY, Peng X. Efficacy of computed tomography features in the differentiation of basal cell adenoma and Warthin tumor in

the parotid gland. Oral Surg Oral Med Oral Pathol Oral Radiol. 2021 Jan 2:4540. doi: 10.1016/j.oooo.2020.12.022. Online ahead of print.PMID: 33478930.

139. Zaccarini DJ, Khurana KK. Incidence of Non - Salivary Gland Neoplasms in Patients with Warthin Tumor : A Study of 73 Cases. Head Neck Pathol. 2020;14(2):412-418. doi:10.1007/s12105-019-01049-7.

140. Zhang D, Li X, Lv L, Yu J, Yang C, Xiong H, Liao R, Zhou B, Huang X, Liu X, Tang Z. Improving the diagnosis of common parotid tumors via the combination of CT image biomarkers and clinical parameters. BMC Med Imaging. 2020 Apr 15;20(1):38. doi: 10.1186/s12880-020-00442-x. PMID: 32293304; PMCID: PMC7161241.

141. Zhao L, Mao Y, Mu J, Zhao J, Li F, Zhang S, Xin X. The diagnostic value of Superb Microvascular Imaging in identifying benign tumors of parotid gland. BMC Med Imaging. 2020 Sep 16;20(1):107. doi: 10.1186/s12880-020-00506-y. PMID: 32938423; PMCID: PMC7493138.
